# Autonomous Search of Radioactive Sources through Mobile Robots

**DOI:** 10.3390/s20123461

**Published:** 2020-06-19

**Authors:** Jianwen Huo, Manlu Liu, Konstantin A. Neusypin, Haojie Liu, Mingming Guo, Yufeng Xiao

**Affiliations:** 1 Robot Technology Used for Special Environment Key Laboratory of Sichuan Province, Southwest University of Science and Technology, Mianyang 621010, China; huojianwen2008@hotmail.com (J.H.); liumanlu@swust.edu.cn (M.L.); liuhaojie_work@163.com (H.L.); GUOMINGMING985@163.com (M.G.); 2 Bauman Moscow State Technical University, Moscow 105005, Russia; neysipin@mail.ru

**Keywords:** autonomous search, radioactive sources, POMDP, measurement error model, mobile robot

## Abstract

The research of robotic autonomous radioactivity detection or radioactive source search plays an important role in the monitoring and disposal of nuclear safety and biological safety. In this paper, a method for autonomously searching for radioactive sources through mobile robots was proposed. In the method, by using a partially observable Markov decision process (POMDP), the search of autonomous unknown radioactive sources was realized according to a series of radiation information measured by mobile robot. First, the factors affecting the accuracy of radiation measurement during the robot’s movement were analyzed. Based on these factors, the behavior set of POMDP was designed. Secondly, the parameters of the radioactive source were estimated in the Bayesian framework. In addition, through the reward strategy, autonomous navigation of the robot to the position of the radiation source was achieved. The search algorithm was simulated and tested, and the TurtleBot robot platform was used to conduct a real search experiment on the radio source Cs-137 with an activity of 37 MBq indoors. The experimental results showed the effectiveness of the method. Additionally, from the experiments, it could been seen that the robot was affected by the linear velocity, angular velocity, positioning accuracy and the number of measurements in the process of autonomous search for the radioactive source. The proposed mobile robot autonomous search method can be applied to the search for lost radioactive sources, as well as for the leakage of substances (nuclear or chemical) in nuclear power plants and chemical plants.

## 1. Introduction

For more than half a century, nuclear energy and nuclear technology have been steadily developed in the world. Nuclear energy plays an important role in optimizing the energy structure, ensuring energy security, promoting pollution reduction and responding to climate change, etc. Radioactive materials have been widely used in the fields of industry, agriculture, national defense, medical treatment and scientific research, which have effectively promoted national production and economic and social development. However, in the process of nuclear energy and nuclear technology application, if a nuclear radiation accident occurs, it will pose a great threat to social security and the country’s political economy, easily causing large-scale casualties and widespread social panic.

According to statistics from the International Atomic Energy Agency (IAEA) [1], as of 31 December 2019, more than 3686 nuclear accidents have been confirmed by the Illegal Traffic Database (ITDB) on theft of nuclear and radioactive materials and other illegal activities. Therefore, it is an important safety issue in the application of nuclear technology to detect the radioactive distribution of radioactive sources in space, and quickly search for and remove nuclear radioactive materials in scattered areas.

Although researchers have been paying attention to this topic for more than two decades, the detection and search of unknown radioactive sources is still a challenging operation in real environment. The main difficulties come from: (1) If the missing or stolen radioactive source is searched by operation personnel, it will increase the operation time and operator’s health risk [2]; (2) if it is searched by a robot, due to the unknown position and direction of the radioactive source and limited perspective of the robot’s observation, the search process is very difficult [3].Therefore, in this paper, an autonomous search algorithm for unknown radioactive sources is designed by using a partially observable Markov decision process (POMDP). The main contributions of this paper are as follows: (1) The robot radiation measurement error model is established, and the factors that affect the radiation measurement results during the movement of the robot are found, which helps the design of the autonomous search algorithm in the later stage. (2) The coordinate, direction of the detection point and detector count of the robot at the current moment are taken as current knowledge, and the posterior probability density function (PDF) of radioactive source parameters is used as the information status in the POMDP. The next action of the robot is selected through the reward strategy of information entropy. (3) The Markov chain Monte Carlo (MCMC) method is used to improve the particle filter to approximate the calculation of PDF, thereby completing the importance sampling of particles.

To study the above topics, this paper is organized as follows: In Section 2, related research in this area is investigated. In Section 3, the error model analysis of robotic radiation measurement is performed. In Section 4, based on the error measurement model, an autonomous search algorithm for unknown radioactive sources is designed. In Section 5, simulation and real experiments are performed. Finally, the conclusions and shortcomings are described.

## 2. Prior Work

In this section, a survey of literature on radioactive source parameter estimation and search strategies is conducted. The search of search strategies is not only about radioactive sources in nuclear physics, but also gas diffusion sources (including biochemistry).

### 2.1. Estimation of Source Parameter

The essence of parameter estimation is to calculate the position and Source Term Parameter of the radioactive source. The traditional method uses measured values of nuclear radiation detectors to determine whether there is a radioactive source in a certain area, and then measures multiple measurement points, and uses the least square method [4,5] or geometric method [5,6,7] to estimate the position and the intensity of the radioactive source. In addition, in [4], the position is correlated with the count rate of the detector; and the change of radiation source position and the deviation between the measured value and the model prediction are also considered. In [5], several probabilistic methods are also provided to estimate the position and intensity of the radioactive source, such as maximum likelihood estimation. In [6], a combined geometric positioning method and sequential probability ratio test are used for radioactive source positioning. Traditional methods rely on the sensitivity of the detector.

In addition, according to the principle of mathematical statistics, radioactive decay occurs randomly, but follows a certain statistical distribution (Poisson distribution or normal distribution), so when estimating the parameters of a radioactive source, the measured value of the detector can be described as a random variable, just like the maximum likelihood estimation method in [5,8]. But when there are three or more radioactive sources at the same time, the maximum likelihood estimation method is not applicable. In addition, the higher SNR thresholds are also considered to be its shortcomings. In [8,9,10,11,12,13], the Bayesian estimation algorithm was used to make up for the shortcomings of the maximum likelihood estimation method. According to Bayesian theory, the posterior probability distribution of the parameter vector of the radioactive source is constructed from the observation data in the radiation field. Therefore, the information of the radioactive source is obtained by solving the posterior probability distribution, which can be approximated and represented by importance sampling [8], progressively corrected importance sampling [11], particle filtering [9,10] and MCMC methods [12,13]. It is worth noting that [12] revealed that the accuracy of the measurement count rate is closely related to the orientation of the detector, which provides a good idea for the error analysis of robotic radiation measurement.

In addition to the above two methods, a Gaussian mixture model is also used to characterize the radiation field and establish a radiation map. Using a logarithmic gradient classifier, the local graph is segmented into several positions of interest to perform radioactive source localization [14]. In the chaotic 3D environment [15], a method of drawing a spatial distribution map of radiation in the environment using a gamma camera was proposed. The source can be effectively found and located according to the spatial distribution map.

### 2.2. Search Strategy

The simplest method for a search strategy is to search in a fixed manner within a specific area. For example, in [7] the UAV was used to detect radioactive materials after the nuclear power plant accident. The UAV moved in a circular trajectory in the air, and estimated the position of the radioactive source as a centroid, which is calculated by using the detected dose of three points. The three-point positioning method has very high requirements on the detector’s response time. If the response time is long, the estimation error will be very large. In [16,17,18,19] the detector was mounted on the UAV to search in a traversal manner. The advantage of this method is that it does not require prior estimation of the radioactive source’s parameters, and the search accuracy was high, but the search efficiency was low. In order to save time and maximize the search efficiency, [19] also proposed the binary search method and the successive approximation search method. The binary search method is suitable for radioactive sources with high activity. Compared with the traversal search algorithm, it was implemented by discarding half of the area at a time, which greatly reduces search time. The successive approximation search method required the activity of the radioactive source to be higher, because it needs to detect a significant change in dose rate at the boundary of the region.

The second method of a search strategy is based on behavior and criteria [20,21,22]. For example, [20] used behavior-based navigation, combining data detected from previously visited areas. This allows the robot to detect safely and effectively. The study in [21] introduced a new behavior-based method for navigating mobile robots in an unknown environment. In this method, each behavior was implemented by a fuzzy controller and executed independently. The authors in [22] constructed a behavior-based control system architecture for the detection of radioactive hot spots under the limitation of sensors. Such methods need to define robot-related behaviors before searching, such as avoiding collisions, detecting radioactive sources and so on. The robot took corresponding actions based on the perceived environmental characteristics and defined criteria until the source item was searched. Such methods are difficult to apply to unknown environments because it is difficult to design behaviors and norms in that conditions.

The third method is as follows. The robot moves randomly or in a fixed manner for a period of time in the search area. With the sensor sensing the radiation information, the source position is calculated by using the source parameter estimation method during this period. Then, combining the methods of information gain [9,10,23], information entropy [24], artificial potential field [25,26], etc., the robot could move to the target point. It is worth noting that in [26], when detecting gamma rays, the incidence angle of the sensor affected the measured radiation intensity. This method required very high accuracy of the source item estimation. If the estimation is not accurate, the robot cannot find the source. The dynamically updated search method [27,28,29,30,31,32] can make up for this shortcoming well. For example, in [27] the dynamic programming method for robot chemical source tracking was proposed. The robot searched for chemical sources based on real-time wind speed and the estimated chemical gas concentration. In [28], in the Bayesian framework, the search efficiency was improved by using the semantics between the detected and recognized gas and the objects in the environment. In addition, the probability of detection and recognition was correlated with the robot’s current position and target distance though using the Markov decision process, to minimize the search time. In [29,30,31,32], POMDP was used to design the gas source search algorithm and the Bayesian frame was used to estimate the gas source parameters. PDF acted as the information state in POMDP, and the gas source search was completed through the reward mechanism and behavior selection. In [29,30] three reward mechanisms were provided: information entropy, Infotaxic II and Bhattacharyya distance. In [31], relative entropy (also known as Kullback–Leibler divergence) was used as a reward for POMDP. The authors in [32] combined the potential energy and the entropy into free energy to be minimized as the reward of POMDP. The studies in [29,30,31,32] provided good ideas for the autonomous search algorithm for radioactive sources in this paper.

This paper uses POMDP to design a method for robots to autonomously search for unknown radioactive sources, but the differences from the above reference are: (1) This paper establishes an error model of the nuclear radiation measurement of the robot during the search process and analyzes the influence of the robot’s speed, positioning accuracy and detection angle on the unknown radioactive source position estimation. (2) In this paper, the speed and angular velocity of the robot are related together as a limited set of actions for POMDP. However, in reference [29,30,31,32] the POMDP behavior set was a single direction indicating behavior, such as {.,↑,→,↓,←}. The limited action set method provided in this paper can make the robot more maneuverable in the search process. (3) The current knowledge is related to the direction of robot movement, rather than a single coordinate and detector count, and the MCMC method was used to improve the particle filter to approximate the posterior, and then complete the importance sampling of the particles.

## 3. Analysis of Robotic Radiation Measurement Model

### 3.1. Radiation Measurement Principle

Assuming that the lost or stolen radioactive source is approximately a point, so only the source activity and spatial location are considered in the study, and the spatial volume is not considered. In homogeneous air, the exposure dose rate of the γ-ray source at distance R is [33]: $\dot{X}=\frac{dX}{dt}=\text{Г}\frac{A}{R^{2}}$. Among them, X is the exposure; Г and A is the exposure dose rate constant and the activity of the point radiation source, respectively; $R^{2}={\left(x_{i}-x_{0}\right)}^{2}+{\left(y_{i}-y_{0}\right)}^{2}$, where $x_{0},y_{0}$ is the source coordinate. The dose equivalent rate $\dot{H}$ (μSv/h) of γ radiation source at distance R can be obtained from Equation (1),

(1) $$\dot{H}=\frac{dH}{dt}=\frac{d\left(wD\right)}{dt}=\frac{d\left(wfX\right)}{dt}=\text{Г}wf\frac{A}{R^{2}}\;,$$

where w is the radiation weighting factor, and the value of photon and electron is 1; D is the absorbed dose; f is the conversion factor for converting exposure into absorbed dose.

The radioactive decay of radioactive materials generally occurs randomly, but within a certain time interval, through the statistics of a large number of atoms, it can be found that the decay process is subject to certain statistical laws. In radiation measurement, the number of radioactive particles emitted by a radioactive source in a unit time can be detected. This process has statistical fluctuations in radioactive counts and obeys the Poisson distribution [9],

(2) $$P\left(C_{pm},\lambda \right)=\frac{\lambda^{C_{pm}}}{C_{pm}!}e^{-\lambda }\;,$$

where $C_{pm}\in N^{+}$ is the count rate per minute ($\min^{-1}$) of the detector, which represents the detected count value within a minute;  λ=ηM, where M is the average value of multiple measurements of N particles generated by the decay of the radioactive source in a certain time interval; η is the efficiency of the detector. $C_{pm}$ can be calculated from Equation (3),

(3) $$C_{pm}=\dot{H}\times Energynumber\;,$$

where Energynumber is energy response constant [5].

### 3.2. Error Analysis of Robotic Radiation Measurement Model

The position of the radiation source is estimated by using Equation (1) after the robot measures multiple points. However, factors such as the detection angle, detection distance, detection time and environmental media [34] bring difficulty to the position estimation. Therefore, it is necessary to find the factors that interfere with the robot’s radiation measurement and reduce the measurement uncertainty during the movement.

**Figure 1:**
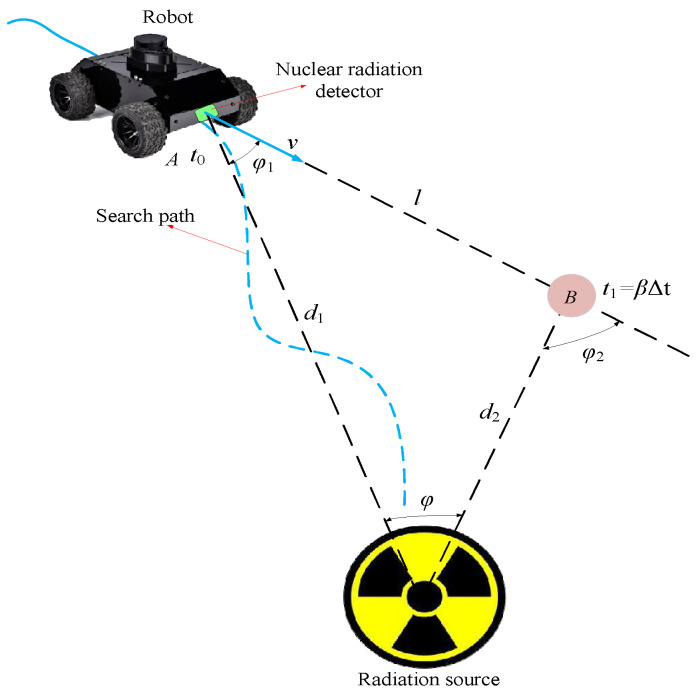
Robot’s radiation measurement model.

Assume that the motion model of the mobile robot is differential motion model, that is:

(4) $$\{\begin{array}{c} \dot{x}=v\cos\theta \\ \dot{y}=v\sin\theta \\ \dot{\theta }=\omega \end{array}\;,$$

where x,y,θ are robot position and direction; v is linear velocity; ω is angular velocity. Assume that the robot is at point A $\;\left(x_{1},y_{1}\right)$ at time $t_{0}$, and moves to point B $\left(x_{2},y_{2}\right)$ at time $t_{1}$ (shown in Figure 1), where $t_{1}=\beta ∆t$. According to the principle of triangle, we can get

(5) $$d_{2}=l\frac{\sin\varphi_{1}}{\sin(\varphi_{2}-\varphi_{1})}\;.$$

Among them, $d_{2}$ is the distance from point B to the radioactive source; l is the distance between point A and point B, $l^{2}={\left(x_{2}-x_{1}\right)}^{2}+{\left(y_{2}-y_{1}\right)}^{2}$; $\varphi_{1}\left(\varphi_{2}\right)$ is the angle between the line connecting the point (A or B) and the radiation source and the direction of the robot’s movement. It should be noted here that the detector is installed directly in front of the robot and there is no relative displacement between the detector and the robot, so the robot’s moving direction is the detection orientation. Robot movement is a continuous process, and $\varphi_{2}$ changes with time, so $\varphi_{2}$ can be approximated by the least squares polynomial form as

(6) $$\varphi_{2}=\sum_{j=0}^{p-1}a_{j}{\left(\beta ∆t\right)}^{j}\;,$$

where $a_{j}$ is polynomial coefficient, $a_{1}=\dot{\varphi }=\frac{v\sin\varphi_{1}}{d_{2}}$, $a_{2}=\frac{1}{2!}\ddot{\varphi }$=$\frac{1}{2}\frac{2v^{2}\sin\varphi_{1}\cos\varphi_{1}}{d_{2}^{2}}={\dot{\varphi }}_{1}^{2}\cot\varphi_{1}$, $\varphi =\varphi_{2}-\varphi_{1}$; p is fitting times; β is time interval coefficient.

According to Equation (5), the error model $∆d_{2}$ of the linear approximation of the distance $d_{2}$ from point B to the radiation source is,

(7) $$∆d_{2}=\frac{\partial d_{2}}{\partial l}∆l+\frac{\partial d_{2}}{\partial \varphi }∆\varphi +\frac{\partial d_{2}}{\partial \varphi_{1}}∆\varphi_{1}=d_{2}\left(\frac{∆l}{l}-∆\varphi_{2}\cot\varphi +∆\varphi_{1}\cot\varphi +∆\varphi_{1}\cot\varphi_{1}\right)\;,$$

where $∆l,∆\varphi_{1},∆\varphi_{2}$ are the errors of $l,\varphi_{1},\varphi_{2}$, respectively. It is not difficult to see from Equation (7) that the positioning error of the radiation source is related to the robot self-positioning, the angle between the line connecting the γ-ray source and the sensor and the direction of the sensor’s movement, and whether other factors are related needs further discussion. Assume that the anticipation error of positioning and incidence angle is zero and they are not related to each other, so

(8) $$\{\begin{array}{c} E\left[\frac{∆d_{2}}{d_{2}}\right]=0\\ \sigma_{∆d_{2}}^{2}=\frac{\sigma_{∆l}^{2}}{l^{2}}+\sigma_{\varphi_{2}}^{2}\cot^{2}\varphi +\left(\cot^{2}\varphi +\cot^{2}\varphi_{1}\right)\sigma_{\varphi_{1}}^{2} \end{array}.$$

Among them, $\sigma_{∆l}^{2},\sigma_{\varphi_{2}}^{2},\sigma_{\varphi_{1}}^{2}$ are the measurement variance of the corresponding parameters, and their value are unknown. The distance l between point A and point B is a function of $x_{1},y_{1},x_{2},y_{2}$. The linear approximation model error ∆l is obtained as $∆l=\frac{\partial l}{\partial x_{1}}∆x_{1}+\frac{\partial l}{\partial y_{1}}∆y_{1}+\frac{\partial l}{\partial x_{2}}∆x_{2}+\frac{\partial l}{\partial y_{2}}∆y_{2}$, so the variance $\sigma_{∆l}^{2}$ is calculated as

(9) $$\sigma_{∆l}^{2}=(\sigma_{∆x_{1}}^{2}+\sigma_{∆x_{2}}^{2})\cos^{2}\theta +(\sigma_{∆y_{1}}^{2}+\sigma_{yx_{2}}^{2})\sin^{2}\theta .$$

According to [35], $\sigma_{∆x_{1}}^{2}=\sigma_{∆y_{1}}^{2}=\frac{4\sigma_{p}^{2}}{N}$, where $\sigma_{p}^{2}$ is the measurement variance of the position sensor, N is the number of measurements. $\;\sigma_{∆x_{2}}^{2}=\sigma_{∆y_{2}}^{2}=\frac{4\sigma_{p}^{2}}{N}\left(1+3\frac{\beta^{2}}{N}\right)$. $\sigma_{\varphi_{1}}^{2}=\frac{4\sigma_{\varphi }^{2}}{N}$, where $\sigma_{\varphi }^{2}$ is the measurement variance of the incident angle between the γ-ray and the sensor. From Equation (6),

(10) $$\sigma_{\varphi_{2}}^{2}=\sigma_{∆a_{0}}^{2}+\sigma_{∆a_{1}}^{2}{\left(\beta ∆t\right)}^{2}+\cdots +\sigma_{∆a_{p}}^{2}{\left(\beta ∆t\right)}^{2p}+\cdots$$

when p=2, combined with the literature [35], Equation (10) can be rewritten as

(11) $$\sigma_{\varphi_{2}}^{2}=\frac{4\sigma_{\varphi }^{2}}{N}+\frac{12\sigma_{\varphi }^{2}}{N^{3}∆t^{2}}{\left(\beta ∆t\right)}^{2}+\sum_{j=2}^{\infty }a_{j}^{2}{\left(\beta ∆t\right)}^{2j}\;.$$

According to the Lagrange multiplier, Equation (11) can be obtained in the final form as

(12) $$\sigma_{\varphi_{2}}^{2}=\frac{8\sigma_{\varphi }^{2}}{N}+\frac{12\sigma_{\varphi }^{2}}{N^{3}∆t^{2}}{\left(\beta ∆t\right)}^{2}\;.$$

In addition, the distance l between point A and point B can be determined according to the robot’s speed v, that is,

(13) l=v(β+N)∆t .

Similarly, $\;\cot\varphi =\frac{d_{2}}{v\sin\varphi_{1}\left(\beta +N\right)∆t}=\frac{1}{\dot{\varphi }\left(\beta +N\right)∆t}$. According to Equations (8)–(14), $\sigma_{∆d_{2}}^{2}$ can be calculated as

(14) $$\sigma_{∆d_{2}}^{2}=\frac{4\left(2+3\frac{\beta }{N^{2}}\right)}{N{\left(\beta +N\right)}^{2}}\left(\frac{\sigma_{p}^{2}}{v^{2}∆t^{2}}+\frac{\sigma_{\varphi }^{2}}{{\dot{\varphi }}^{2}∆t^{2}}\right)+\frac{4\sigma_{\varphi }^{2}}{N}\cot^{2}\varphi_{1}\;.$$

During the measurement, $\sigma_{∆d_{2}}^{2}$ needs to be minimized, so use the following formula to get the value of β:

(15) $$\frac{d\left(\frac{4\left(2+3\frac{\beta }{N^{2}}\right)}{N{\left(\beta +N\right)}^{2}}\right)}{d\beta }=0\to \beta =\frac{2N}{3}\;.$$

Bring Equation (15) into Equation (14), so

(16) $$\sigma_{∆d_{2}}^{2}=\frac{24}{5N^{3}}\left(\frac{\sigma_{p}^{2}}{v^{2}∆t^{2}}+\frac{\sigma_{\varphi }^{2}}{{\dot{\varphi }}^{2}∆t^{2}}\right)+\frac{4\sigma_{\varphi }^{2}}{N}\cot^{2}\varphi_{1}.$$

From Equation (16), we can see that the robot’s positioning accuracy of the radioactive source during the movement is affected by the number of measurements, the measurement accuracy of position sensor, the angle between the line connecting the gamma ray and the measurement sensor and the direction of the sensor’s movement, and the speed of the robot. By analyzing the factors that affect the positioning of the radioactive source, it provides help for the following mobile robot radioactive source positioning strategies.

## 4. Unknown Radioactive Source Search Strategy

### 4.1. Search Strategy

The position of the radioactive source is initially unknown for a searching robot. Based on its own perception of the surrounding environment, by using the local information for path planning, the robot could finally find the unknown radioactive sources. Furthermore, since radioactive source is strictly controlled items, if it gets lost, its number is generally known. Therefore, in this paper, we just consider the case of a single lost radioactive source.

Robot autonomous search strategy was designed using POMDP [36] in this paper. In POMDP model, the robot needs to collect environmental information (observation values) through sensors to update its credibility on the current state, which is the basis for the robot’s decision on the next action choice. According to Equations (3) and (4), the observation sequence of the sensor when the robot moves to the target point is established, that is, $z_{1:k}=\left\{z_{1},z_{2},z_{3},\cdots ,z_{k}\right\}$, where $z_{k}={\left(x_{k},y_{k},\theta_{k},C_{pmk}\right)}^{T}$ represents the measurement value $C_{pmk}\;$ obtained by the robot at a certain position ($x_{k},y_{k}$) and in a certain direction $\theta_{k}$. Assume the parameter vector I of the unknown radioactive source is $I={\left(x_{0},y_{0},\theta_{0},I\right)}^{T}$, where $x_{0},y_{0}$ is the coordinate of the plane position, $\theta_{0}$ is the included angle of the radiation source with respect to the movement direction of the starting point of the robot, I is the activity of radioactive source and the unit is Bq. According to Bayes’ rule, the likelihood function of the observation sequence $z_{1:k}$ is,

(17) $$p\left(z_{1:k}|I\right)=\overset{k}{\underset{i=1}{\prod }}p\left(z_{i}|I\right)\;,$$

among them, $p\left(z_{i}|I\right)=P\left(C_{pmi},\lambda_{i}\right)$. Furthermore, according to Bayesian theory, after obtaining the observation sequence $z_{1:k\;}$ in the radiation field, the posterior probability distribution function of the radiation source’s parameter vector I can be obtained as $p\left(I|z_{1:k}\right)$. Since the searching action of radioactive source is a gradual process, the asymptotic calculation formula of the posterior probability distribution function is as follows:

(18) $$p\left(I|z_{1:k}\right)=\frac{p\left(z_{i}|I,z_{1:i-1}\right)p\left(I|z_{1:i-1}\right)}{p\left(z_{i}|z_{1:i-1}\right)},1\leq i\leq k\;,$$

Then, according to Equation (18), the information state of discrete time k+1 can be obtained as

(19) $$p\left(I|z_{1:k+1}\right)=\frac{p\left(z_{i+1}|I\right)p\left(I|z_{1:k}\right)}{\int^{\text{​}}p\left(z_{i+1}|I\right)p\left(I|z_{1:k}\right)dI}\;,\;1\leq i\leq k\;.$$

In addition, when k=0, p(I) is the prior distribution of the source’s parameters. At first, it was thought that unknown radioactive sources could be located anywhere in the search area and could be set to be evenly distributed. Next, after making certain judgments based on local information, the robot needs to continue moving to make further observation records until the radioactive source is searched. Then the robot’s finite action set is defined as ***A*** = {(*v*, ∆*θ*),(−*v*, ∆*θ*),(*v*, −∆*θ*),(−*v*, −∆*θ*),(*v*, 0),(−*v*, 0),(0, ∆*θ*),(0, −∆*θ*),(0, 0)}, where ∆θ is the robot’s rotation angle within the time step ∆t, which is determined by the angular velocity ω, that is, ∆θ=ω∆t. Thus, the finite action set can be simplified as ***A*** = {(*v*, *ω*),(−*v*, *ω*),(*v*, −*ω*),(−*v*, −*ω*),(*v*, 0),(−*v*, 0),(0, *ω*),(0, −*ω*),(0, 0)} .Then the position and direction of the robot at time k+1 can be obtained from Equation (20),

(20) $$\{\begin{array}{c} x_{k+1}=x_{k}+∆tv\cos\theta_{k}\\ y_{k+1}=y_{k}+∆tv\sin\theta_{k}\\ \theta_{k+1}=\theta_{k}+∆t\omega \end{array}\;.$$

According to Equation (20), the measurement value $z_{k+1}$ of the sensor at time k+1 depends on the position and direction at time k and the selected action a (a∈A). Within each time step ∆t, the robot should move in the direction where the expected count rate is maximum. Therefore, this paper uses information entropy to describe the reward of action a. Then the robot’s information entropy during the search process changes to [37],

(21) $$D\left(a_{k}\right)=-Pr_{k}S_{k}+\left(1-Pr_{k}\right)\left(E\left(S_{k+1}\right)-S_{k}\right)\;,$$

where $S_{k}$ is the information entropy of the radioactive source, that is, $S_{k}=\int^{\text{​}}p\left(I|z_{1:k}\right)\log p\left(I|z_{1:k}\right)dI$ and $Pr_{k}\;$ can be calculated using the kernel density estimation method [38]. The physical meaning of Equation (21) is explained in [37]. The first term on the right side of the formula indicates that the robot has found the radioactive source at time k+1, while the second term indicates that the robot has not yet searched the source at time k+1, and needs further searching until the source is found, so $S_{k+1}=0$, and in the search process, only the second term of Equation (21) is used to represent the reward. According to the Equation (2), the expectation of the Shannon entropy $S_{k+1}$ of measured value $z_{k+1}$ at the time k+1 can be obtained as

(22) $$E\left(S_{k+1}\right)=\overset{C_{pm}}{\underset{C_{pm}=0}{\sum }}P\left(C_{pm},\lambda \right)S_{k+1}=\overset{C_{pm}}{\underset{\lambda_{k+1}=0}{\sum }}p\left(z_{k+1}|I\right)\int^{\text{​}}p\left(I|z_{1:k+1}\right)\log p\left(I|z_{1:k+1}\right)dI\;.$$

Then, while the robot is looking for a radioactive source, according to the optimal strategy $\pi_{k}{}^{*}$, $D\left(a_{k}\right)$ is minimized, that is,

(23) $$\pi_{k}{}^{*}=\underset{a_{k}\in A}{argmin}D\left(a_{k}\right).$$

### 4.2. Parameter Estimation of Radioactive Sources

Using a mobile robot to search for radioactive sources is a gradual process, and the radioactive source parameters are gradually estimated according to the search strategy of Section 4.1. However, in the search strategy, the posterior probability distribution function is difficult to obtain analytically. Therefore, the MCMC method is used to improve the particle filter to approximate the posterior probability distribution function. The basic idea of the particle filtering algorithm is to use a group of n particles with their own weights $\left\{I_{k}^{i},w_{k}^{i}\right\},i=1,\cdots ,n$ to approximate the posterior probability density $\;p\left(I|z_{1:k}\right)$ of the radioactive source parameter I. In addition, the weight of the particles satisfies $\overset{n}{\underset{i=1}{\sum }}w_{k}^{i}=1$. Then the posterior probability density is approximately as follows:

(24) $$p\left(I|z_{1:k}\right)\approx \overset{n}{\underset{i=1}{\sum }}w_{k}^{i}\delta \left(I-I_{k}^{i}\right)\;,$$

where δ(·) is a Dirac function. Based on this approximation, complex integration operations can be converted into sum operations, such as $S_{k}\approx -\overset{n}{\underset{i=1}{\sum }}w_{k}^{i}\log w_{k}^{i}$. Assume that the importance sampling density is $q\left(I|z_{1:k}\right)$, the prior probability transfer distributionis is chosen as $q\left(I_{k}|I,z_{1:k}\right)=p\left(I_{k}|I_{k-1}\right)$, then according to the first-order Markov hypothesis, the unnormalized weight of each particle at time k can be obtained as,

(25) $$w_{k}^{*i}=w_{k-1}^{i}\;p\left(z_{k}|I_{k}^{i}\right)=w_{k-1}^{i}P\left(C_{pmk},\lambda_{i}\right).$$

Among them, $C_{pm}$ is the radiation dose counting rate detected by the detector at time k, $\lambda_{i}$ is the estimated value of the radiation intensity of the *i*-th particle at the observation point $\left(x_{k},y_{k}\right)$. The normalized weight of each particle is $w_{k}^{i}=\frac{w_{k}^{*i}}{\sum_{j=1}^{n}w_{k}^{*j}}$, so we can get a set of weighted particles. Then the estimated values of the radiation source’s parameters are obtained according to Equation (24).

However, in particle filtering, due to frequent resampling, the particles will lack diversity, mainly concentrated on some particles with higher weight. In this paper, a valid number of samples $N_{th}$ is set as the threshold. When the weight value $\overset{n}{\underset{i=1}{\sum }}w_{k}^{i}$ of the current particle set is less than $N_{th}$, random resampling is performed by the roulette selection method, and a little noise is added to the particle set after sampling. If without resampling, the particle set is retained directly. In order to improve the diversity of the obtained particle set, the resampled particles are continuously filtered by using the Metropolis–Hastings sampling algorithm [39], so that the sampling points are gradually moved to the central region of the posterior probability distribution. That is, taking $I_{k-1}^{i}$ as the mean and $\tau^{2}$ as the importance sampling function for variance $q\left(I_{k}|I_{0:k,}z_{1:k}\right)$, then $q\left(I_{k}|I_{0:k,}z_{1:k}\right)=N\left(I_{k-1}^{i},\tau^{2}\right)$. New particle $I_{0:k}^{i*}$ is extracted from the importance function $q\left(I_{k}|I_{0:k,}z_{1:k}\right)$, and new particles are accepted with a probability value $\alpha \left(I_{0:k}^{i*}\right)=\min\left\{1,\frac{p(z_{1:k}|I_{0:k}^{i*})}{p(z_{1:k}|I_{0:k}^{i})}\right\}$ until a new particle after the k-th observation is obtained.

## 5. Experimental Analysis and Discussion

### 5.1. Simulation Experiment and Analysis

The simulation preliminarily verified the feasibility of autonomous search of the radioactive source by the robot. The simulation assumptions are as follows:

1. Two-dimensional barrier-free space. The size is 200 m × 200 m;
2. Fixed static radioactive source. $I={\left(140\;m,160\;m,0.852\;\mathrm{rad},2.94\times 10^{8}\;\mathrm{Bq}\right)}^{T}$. The maximum measured value is specified when the robot movement direction θ=0.852 rad;
3. Generate count measurements according to Equation (3) and Poisson distribution noise, where $\;Energynumber=100\;,\text{Г}=2.5\times 10^{-7},w=1,f=30$;
4. The measured value of environmental background radiation count is 1 cps;
5. The calculation method of $Pr_{k}$ refers to the method of [29].

According to the robot’s differential motion model (4), within the duration ∆t=1 s, the robot’s pose at the time k+1 is $\left(x_{k},y_{k},\theta_{k}\right)$, the initial pose of the robot is (30, 20, 0), and the robot’s motion set is ***A***. Combining the factors that affect the positioning accuracy of the radioactive source in Section 3, simulation experiments prove the performance of the algorithm from different schemes.

(1) Algorithm parameters: The number of particles is  n=5000, the initialized particle information $x_{i},y_{i}$ are random numbers in the range of [0, 200], and $I_{i}$ is a random number in the range of $\left[3\times 10^{6},1\times 10^{9}\right]$. The robot’s linear velocity v=5 m/s, angular velocity ω=0.1 rad/s. Within the duration ∆t, the measurement times N are 1, 5, 10, 15, 20 respectively. The condition for the end of the algorithm is that the estimated position error of the radioactive source is less than 1 m (x≤1 m and y≤1 m).

**Figure 2:**
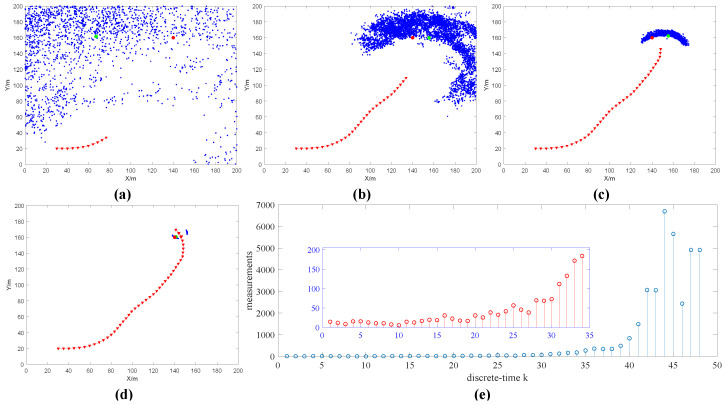
The estimation results of radioactive sources when the number of measurements N=1 (**a**) k=20 , (**b**) k=30, (**c**) k=40, (**d**) k=48. In the figure, the blue dot indicates existing particle positions, the green circle indicates the estimated position of the radioactive source, the red circle indicates the true position of the radioactive source, the red line indicates the robot search path and the red triangle indicates the position of the robot. (**e**) Shows the measured value $z_{k}$ obtained during the source search.

When N=1, the simulation results are shown in Figure 2. Figure 2a–d shows the estimated position of the radiation source (represented by green circles), the position of the robot (represented by triangles) and the search trajectory of the robot at time k=11, k=30, k=40, k=48, respectively. When the robot is at the starting point (30, 20), the observation value $z_{1}$ is obtained according to simulation assumption (3) (as shown in Figure 2e). Combining the observation value $z_{1}$ and the particle information $\left(x_{i},y_{i},I_{i}\right)$, the normalized weight $w_{1}^{i}$ of each particle is calculated. The position and size of the radioactive source are estimated by bringing the normalized weight $w_{1}^{i}$ and particle information $\left(x_{i},y_{i},I_{i}\right)$ into formula (24), and the information entropy is further obtained through Equations (21), (22) and (25). The information entropy is used to select the next movement behavior of the robot. Then, the particle information is updated with the effective sample number $N_{th}=2n/3,$ position variance $\tau_{p}^{2}=50/i,$ intensity variance $\tau_{I}^{2}=4.5\times 10^{7}/i$ and acceptance probability $\alpha \left(I_{0:1}^{i*}\right)$. Finally, the algorithm conditions are judged. If the conditions are met, the algorithm is terminated. If the conditions are not met, the robot moves according to the selected behavior, and then observes, until the conditions are met. The simulation results of measurement times N=5, N=10, N=15, N=20 are shown in Figure S1 (Supplemental Materials 1), Figure S2 (Supplemental Materials 2), Figure S3 (Supplemental Materials 3) and Figure S4 (Supplemental Materials 4).

The error statistics of different measurement times within ∆t are shown in Table 1. It can be seen in Table 1 that as the number of measurements increases, the accuracy of the radioactive source intensity estimation is higher. From Figure 2e and Figures S1–S4, it can be found that the greater the number of measurements, the smaller the random error of the observed value. Due to the influence of the robot linear velocity and angular velocity, as the number of measurements increases, the iteration number k decreases to 36 and does not change.

**Figure 3:**
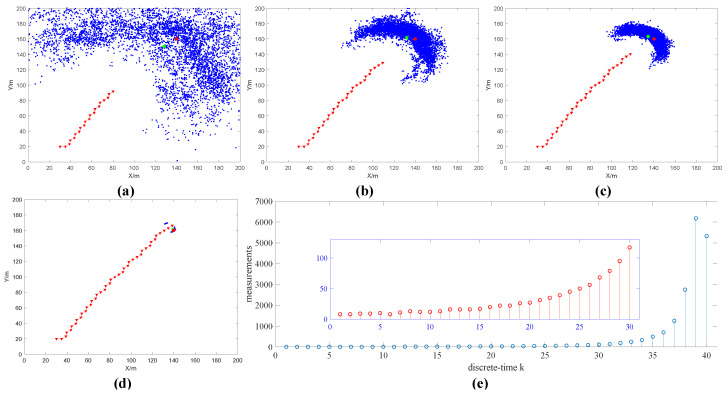
The estimation results of radioactive sources when the angular velocity ω=0.7 rad/s (**a**) k=20, (**b**) k=30, (**c**) k=33 and (**d**) k=40. (**e**) Shows the measured value $z_{k}$ obtained during the search of the radioactive source.

(2) Algorithm parameters: The number of particles n=5000, the robot’s linear velocity v=5 m/s, the angular velocity ω=0.1 rad/s. The number of measurements within ∆t is N=20. The angular velocity of the robot ω=0.1 rad/s, ω=0.3 rad/s, ω=0.5 rad/s, ω=0.7 rad/s, ω=0.9 rad/s. The condition for the end of the algorithm is that the estimated position error of the radioactive source is less than 1 m (x ≤ 1 m and y ≤ 1 m). The simulation results are shown in Figure 3 when ω=0.7 rad/s. The error statistics at different angular velocities are shown in Table 2. As the angular velocity increases, the number of iterations k decreases first and then increases. It is because as the angular velocity increases, the robot’s rotation amplitude increases, which affects the observation value and particle weight, thus affecting the robot’s behavior choice. It can be seen from Figure 3a–d and Figures S5–S7 (Supplemental Materials 5–7) that as the angular velocity increases, the robot search trajectory shows an oscillating trend.

When the number of measurements and the angular velocity of the robot are constant within the duration ∆t, as the robot linear velocity increases during simulation, the number of iterations decreases and the error of the estimated radioactive source remains basically unchanged. However, in the actual process, the robot linear velocity increases. Within constant duration ∆t, the number of measurements should be gradually reduced. It is because in the actual test process the sensor needs a response time, but the simulation process does not. In addition, when the number of measurements, the linear velocity and the angular velocity of the robot are constant within ∆t, the larger the positioning error of the position sensor, the greater the cumulative position error according to Equation (20). Therefore, the observation value obtained by Equation (3) is smaller, resulting in a larger weight value for each particle, so that the estimated position and size of the radiation source are larger.

### 5.2. Real Experiment and Analysis

**Figure 4:**
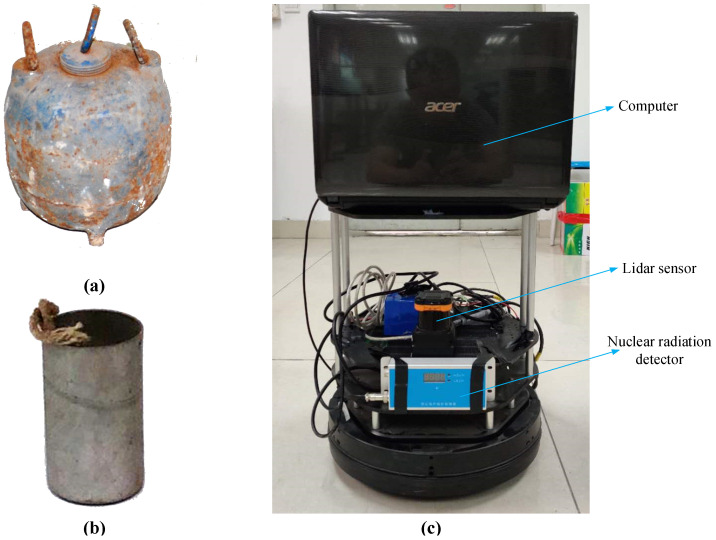
Physical picture of radioactive source and robot system. (**a**) Lead can. (**b**) Radioactive source. (**c**) Robot system.

To test the effectiveness of the search algorithm, the experimental design is as follows:

1. The energy of the gamma rays generated when the Cs-137 radioactive source (as shown in Figure 4b), with 37 MBq activity stored in the lead (as shown in Figure 4a), is 0.662 MeV.
2. The parameters of the G-M radiation detector are as follows: Measurement range—0.01–5000 uSv/h, energy response—40KeV–3 MeV, sensitivity—≥3000 cpm/mR/h, relative error: ≤15%. The five sides of the cuboid are aluminum alloy shells, and the front panel material is plastic.
3. Use the TurtleBot robot as the experimental search robot, as shown in Figure 4c. The robot is equipped with an Acer notebook, and the notebook is installed with the Ubuntu + ROS system.
4. The hokuyo UTM-30 lidar sensor is used to establish an environment map in advance, that is, the environment map already exists during the radioactive source search experiment.
5. Obtain the robot’s coordinates by reading the robot’s odometer information.
6. Equation (1) is simplified as $\dot{H}=\Upsilon \frac{A}{R^{2}}$, and k is constant. The dose equivalent rate measured by the nuclear radiation detector at a distance of 1 m from the radioactive source is 10.45 uSv/h and thus $\Upsilon =2.824\times 10^{-8}$.
7. According to literature [5], for Cs-137 with an average γ-ray energy of 0.662 MeV, the energy response constant is 12,200 cpm/μSv/h.
8. The position coordinate of the radioactive source in the experiment is (6.4 m, 4.0 m, 0.5586 rad), the initial position of the robot is (0, 0, 0), and the number of particles is n=1000.
9. Due to the control of radioactive sources in universities, the experiment is conducted in 12 m × 8 m indoors.

**Figure 5:**
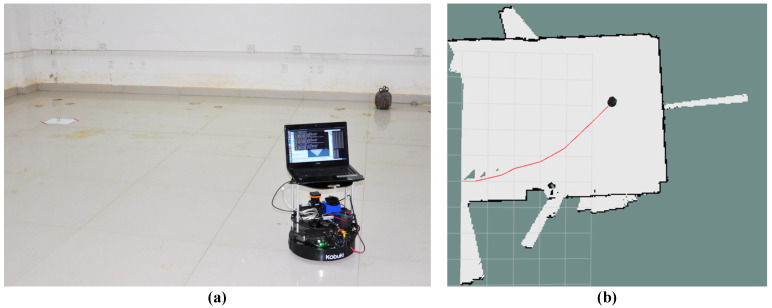
Real experiment. (**a**) Work scene graph of the robot searching for the radioactive source, (**b**) when N=2, v=0.2 m/s, ω=0.1 rad/s, the map and trajectory map of the robot while automatically searching the radioactive source.

Combining the factors that affect the positioning accuracy of the radioactive source in Section 3 and the results of the reference simulation experiments, the angular velocity of the robot in the real experiment is constant ω=0.1 rad/s; the linear velocity of the robot is v=0.1 m/s, v=0.15 m/s,  v=0.2 m/s respectively; the number of measurements in the range of duration ∆t=1s are N=1,N=2,N=3. A total of 9 sets of experiments were conducted. When the estimated position error of the radioactive source satisfies the conditions of x ≤ 0.3 m and y ≤ 0.3 m each time, the robot ends the search. The working scene of the robot autonomously searching for the radioactive source is shown in Figure 5a. The trajectory of autonomous search of the robot (N=2,v=0.2 m/s) is shown in Figure 5b. The results of the whole experiments are shown in Figure S8 (Supplemental Materials 8) and the error statistics are shown in Table 3.

It can be seen from Figure S8 and Table 3 that when the number of measurements is constant, the estimated error of the radioactive source coordinates increases with the increase of the robot linear velocity. This is because in nuclear radiation measurements the G-M tube detector has a response time of tens of milliseconds (some G-M tubes even require a response time of a few seconds). When the response time is constant, the greater the linear velocity of the robot, the greater the distance the robot moves during measurement and the greater the positioning error. When the robot linear velocity is constant, the number of measurements increases and the accuracy of estimating the position of the radiation source also increases. Due to the limitation of the detector’s response time, the number of measurements within the duration ∆t cannot be increased indefinitely. In addition, it can be seen from the experimental results that the method proposed in this paper is not suitable for estimating the activity of radioactive sources.

## 6. Conclusions

In this paper, a method for autonomous search of radioactive sources by mobile robots was proposed. The next behavior of the robot was chosen through POMDP based on the locally observed information. In the Bayesian framework, the improved MCMC method is used to estimate the parameters of the radioactive source, and the robot’s behavior selection is driven by the reward of information entropy. In addition, this paper also analyzes the factors that affect the accuracy of the radiation measurement during the movement of the robot, and designs the behavior set of POMDP according to these factors. Simulation and experimental results show that the algorithm has excellent performance in a barrier-free environment. The estimated accuracy of the radioactive source is related to the behavior of the robot, the number of measurements, the accuracy of the position sensor and the angle of incidence of the radiation and the sensor. In the future, we will study the autonomous search of radioactive sources in obstacle environments, and consider the case where there are multiple radioactive sources or a coordinated search using multiple land robots or a combination of air and land robots.

## Figures and Tables

**Table 1:** Error of radioactive source estimation under different measurement times.

Number	Iterations	Robot Final Position	Estimated Source Coordinates	Error	Estimated Source Size	Error
N=1	k=48	(146.56, 161.06)	(140.16, 159.99)	(0.16, 0.01)	3.11 × 10^8^	1.74 × 10^7^
N=5	k=42	(142.91, 155.04)	(140.98, 160.31)	(0.98, 0.31)	2.97 × 10^8^	3.11 × 10^6^
N=10	k=38	(137.60, 152.33)	(139.45, 159.58)	(0.55, 0.42)	2.86 × 10^8^	8.23 × 10^6^
N=15	k=37	(135.90, 153.19)	(139.45, 159.33)	(0.55, 0.67)	2.89 × 10^8^	5.33 × 10^6^
N=20	k=37	(135.98, 152.46)	(139.4, 159.79)	(0.6, 0.21)	2.90 × 10^8^	4.32 × 10^6^

**Table 2:** Errors of radioactive source estimates at different angular velocities.

Angular Velocity	Iterations	Robot Final Position	Estimated Source Coordinates	Error	Estimated Source Size	Error
ω=0.1	k=37	(135.98, 152.46)	(139.4, 159.79)	(0.6, 0.21)	2.90 × 10^8^	4.32 × 10^6^
ω=0.3	k=36	(137.34, 151.56)	(140.89, 159.05)	(0.89, 0.95)	2.93 × 10^8^	1.30 × 10^6^
ω=0.5	k=36	(135.28, 154.00)	(139.07, 160.19)	(0.93, 0.19)	2.87 × 10^8^	7.03 × 10^6^
ω=0.7	k=40	(138.68, 166.29)	(139.91, 160.01)	(0.09, 0.01)	2.92 × 10^8^	2.45 × 10^6^
ω=0.9	k=45	(129.91, 167.67)	(140.33, 159.92)	(0.33, 0.08)	3.02 × 10^8^	7.90 × 10^6^

**Table 3:** Error of radioactive source estimation in the real experiment.

Number	Velocity	Iterations	Robot Final Position	Estimated Source Coordinates	Error	Estimated Source Size	Error
N=1	v=0.1	k=16	(7.25, 2.28)	(6.24, 4.05)	(0.16, 0.05)	3.03 × 10^7^	6.7 × 10^6^
	v=0.15	k=15	(6.65, 2.56)	(6.44, 3.84)	(0.04, 0.16)	3.47 × 10^7^	2.3 × 10^6^
	v=0.2	k=15	(6.18, 3.25)	(6.19, 3.74)	(0.21, 0.26)	2.53 × 10^7^	1.17 × 10^7^
N=2	v=0.1	k=13	(5.17, 3.2)	(6.37, 3.97)	(0.03, 0.03)	3.31 × 10^7^	3.9 × 10^6^
	v=0.15	k=13	(5.42, 2.77)	(6.47, 3.94)	(0.07, 0.06)	2.93 × 10^7^	7.7 × 10^6^
	v=0.2	k=14	(5.77, 3.14)	(6.27, 3.87)	(0.13, 0.13)	3.01 × 10^7^	6.9 × 10^6^
N=3	v=0.1	k=12	(5.29, 2.08)	(6.42, 4.03)	(0.02, 0.03)	3.88 × 10^7^	1.8 × 10^6^
	v=0.15	k=12	(5.13, 2.25)	(6.34, 3.99)	(0.06, 0.01)	3.44 × 10^7^	2.6 × 10^6^
	v=0.2	k=12	(4.55, 3.36)	(6.54, 4.1)	(0.14, 0.1)	2.97 × 10^7^	7.3 × 10^6^

## Supplementary Materials

The following are available online at https://www.mdpi.com/1424-8220/20/12/3461/s1. Figure S1: The estimation results of radioactive sources when the number of measurements N=5 and angular velocity ω=0.1 rad/s. Figure S2: The estimation results of radioactive sources when the number of measurements N=10 and angular velocity ω=0.1 rad/s. Figure S3: The estimation results of radioactive sources when the number of measurements N=15 and angular velocity ω=0.1 rad/s. Figure S4: The estimation results of radioactive sources when the number of measurements N=20 and angular velocity ω=0.1 rad/s. Figure S5: The estimation results of radioactive sources when the number of measurements N=20 and angular velocity ω=0.3 rad/s. Figure S6: The estimation results of radioactive sources when the number of measurements N=20 and angular velocity ω=0.5 rad/s. Figure S7: The estimation results of radioactive sources when the number of measurements N=20 and angular velocity ω=0.9 rad/s. Figure S8: Real experiment results.
