# Supplementary material for: Autonomous Search of Radioactive Sources through Mobile Robots
Source: Sensors (Basel). 2020 Jun 19;20(12):3461. doi: 10.3390/s20123461 (PMC7349192; doi:10.3390/s20123461)
Supplement: Supplementary file 1 [file sensors-20-03461-s001.pdf]

Supplementary Materials (including eight figures)

Autonomous search of radioactive sources through mobile robots

Supplementary Materials 1: The estimation results of radioactive sources when the number of measurements $\boldsymbol{N = 5}$ and angular velocity $\boldsymbol{\omega = 0.1 rad/s}$.

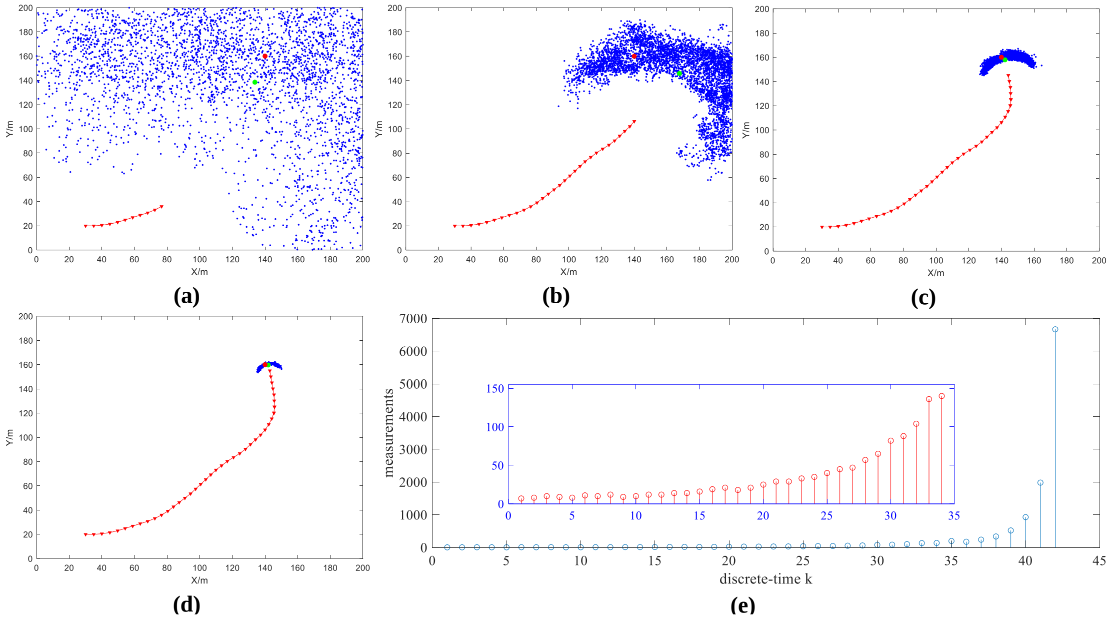

Figure S1. The estimation results of radioactive sources when the number of measurements $N = 5$ and angular velocity $\omega = 0.1 rad/s$ (**a**) $k = 11$, (**b**) $k = 30$, (**c**) $k = 40$, (**d**) $k = 42$. Figure (**e**) shows the measured value $\boldsymbol{z}_k$ obtained during the search of the radioactive source.

Supplementary Materials 2: The estimation results of radioactive sources when the number of measurements $\boldsymbol{N = 10}$ and angular velocity $\boldsymbol{\omega = 0.1 rad/s}$.

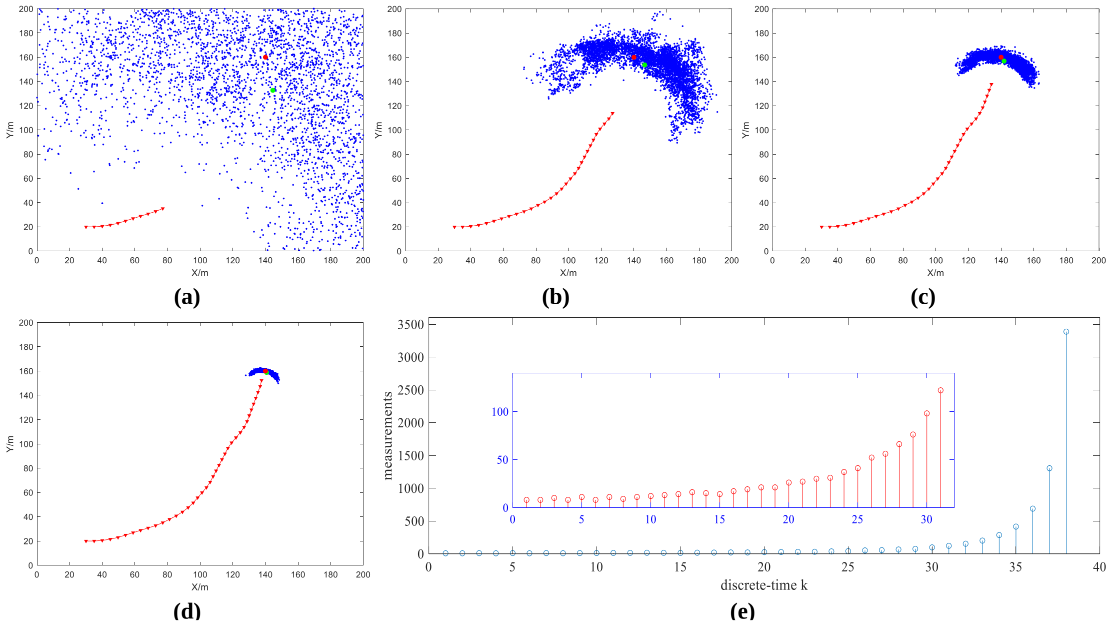

Figure S2. The estimation results of radioactive sources when the number of measurements $N = 10$ and angular velocity $\omega = 0.1 rad/s$ (**a**) $k = 11$, (**b**) $k = 30$, (**c**) $k = 35$, (**d**) $k = 38$. Figure (**e**) shows the measured value $\boldsymbol{z}_k$ obtained during the search of the radioactive source.

Supplementary Materials 3: The estimation results of radioactive sources when the number of measurements $N = 15$ and angular velocity $\omega = 0.1 rad/s$.

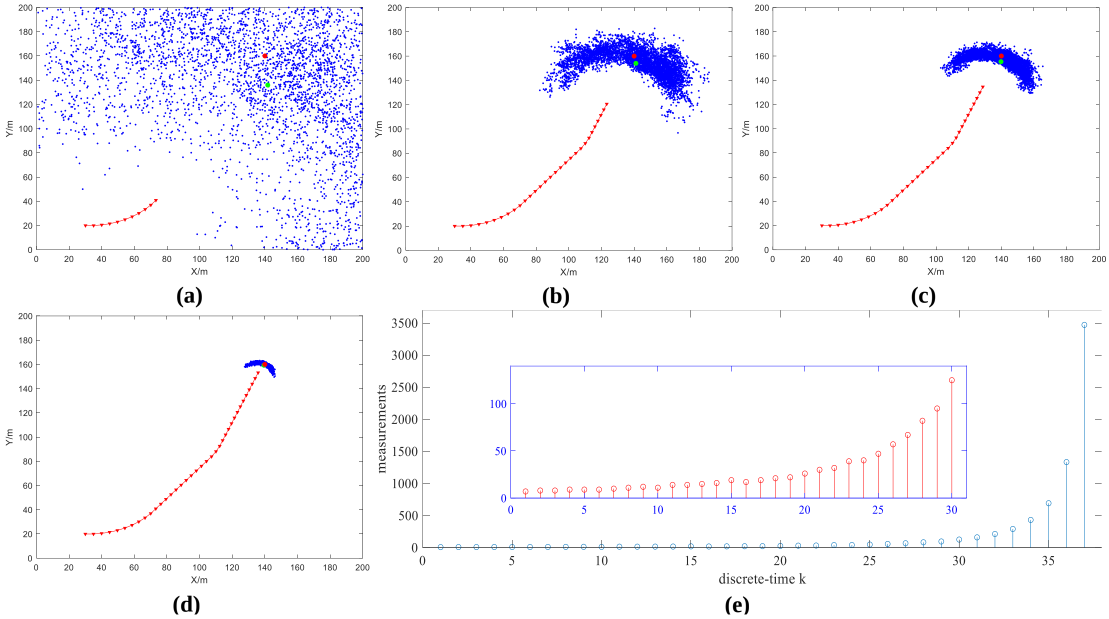

Figure S3. The estimation results of radioactive sources when the number of measurements $N = 15$ and angular velocity $\omega = 0.1 rad/s$ (**a**) $k = 11$, (**b**) $k = 30$, (**c**) $k = 33$, (**d**) $k = 37$. Figure (**e**) shows the measured value z_k obtained during the search of the radioactive source.

Supplementary Materials 4: The estimation results of radioactive sources when the number of measurements $N = 20$ and angular velocity $\omega = 0.1 rad/s$.

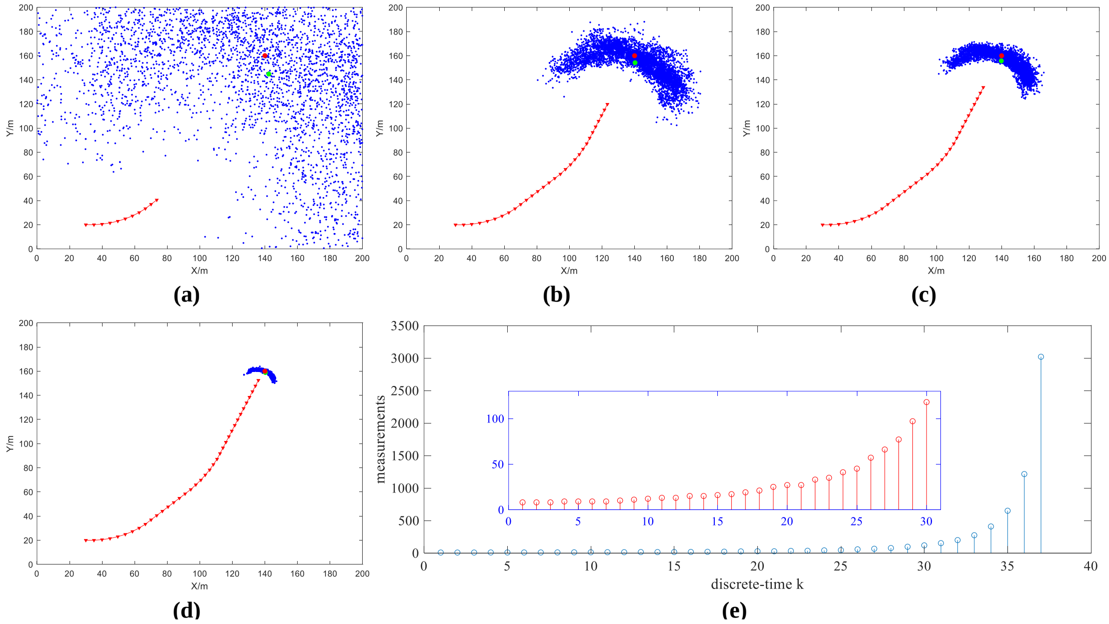

Figure S4. The estimation results of radioactive sources when the number of measurements $N = 20$ and angular velocity $\omega = 0.1 rad/s$ (**a**) $k = 11$, (**b**) $k = 30$, (**c**) $k = 33$, (**d**) $k = 37$. Figure (**e**) shows the measured value z_k obtained during the search of the radioactive source.

Supplementary Materials 5: The estimation results of radioactive sources when the number of measurements $N = 20$ and angular velocity $\omega = 0.3 rad/s$.

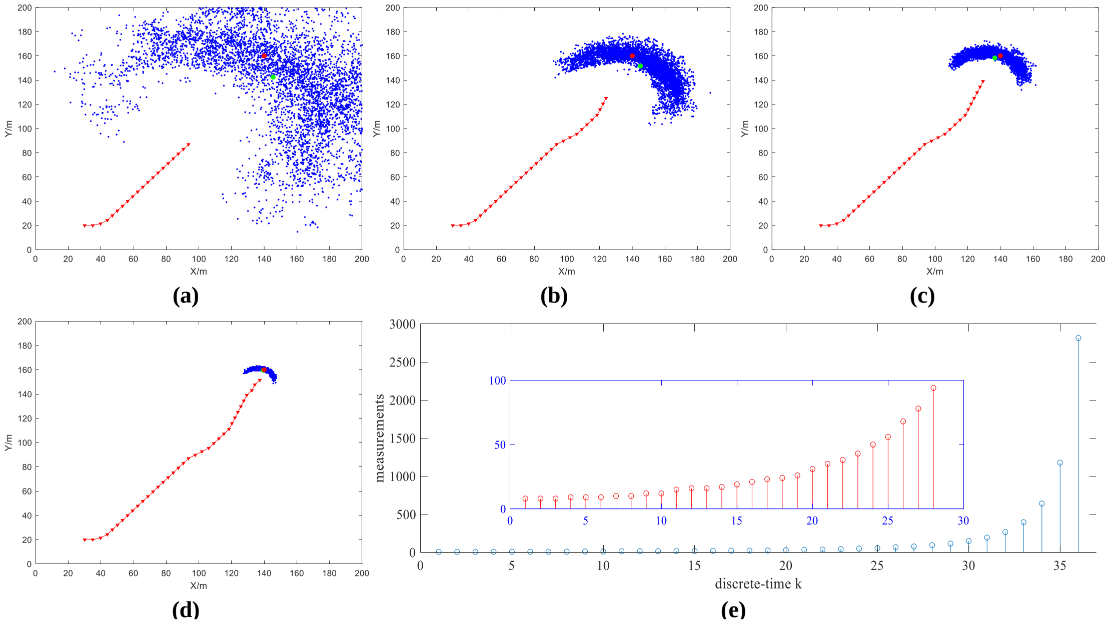

Figure S5. The estimation results of radioactive sources when the number of measurements $N = 20$ and angular velocity $\omega = 0.3rad/s$ (**a**) $k = 20$, (**b**) $k = 30$, (**c**) $k = 33$, (**d**) $k = 36$. Figure (**e**) shows the measured value z_k obtained during the search of the radioactive source.

Supplementary Materials 6: The estimation results of radioactive sources when the number of measurements $N = 20$ and angular velocity $\omega = 0.5 rad/s$.

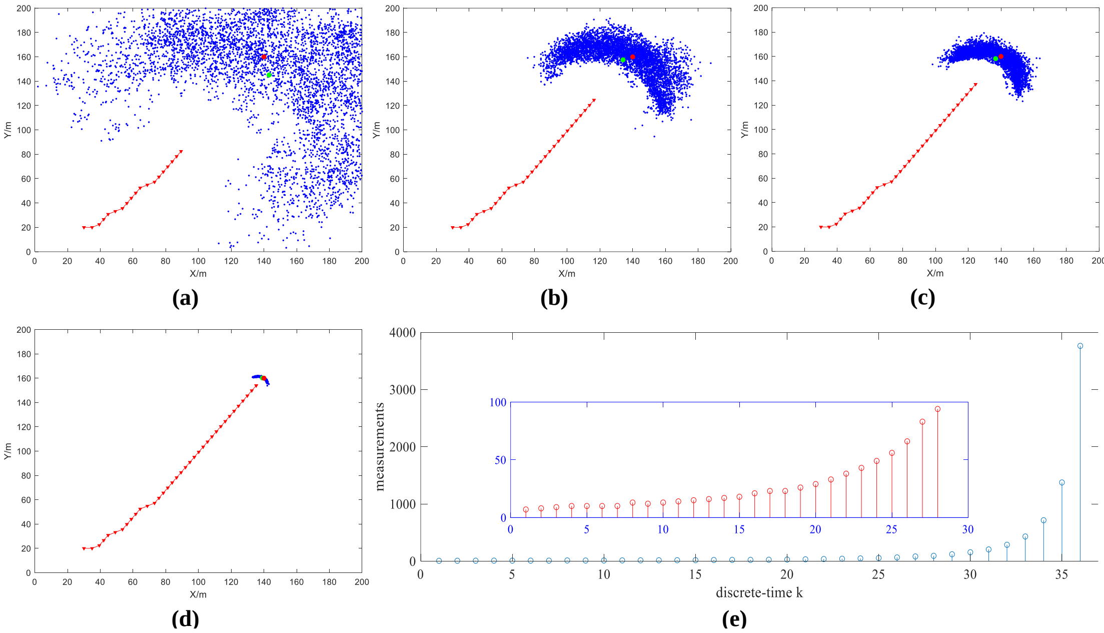

Figure S6. The estimation results of radioactive sources when the number of measurements $N = 20$ and angular velocity $\omega = 0.5rad/s$ (**a**) $k = 20$, (**b**) $k = 30$, (**c**) $k = 33$, (**d**) $k = 36$. Figure (**e**) shows the measured value z_k obtained during the search of the radioactive source.

Supplementary Materials 7: The estimation results of radioactive sources when the number of measurements $N = 20$ and angular velocity $\omega = 0.9rad/s$.

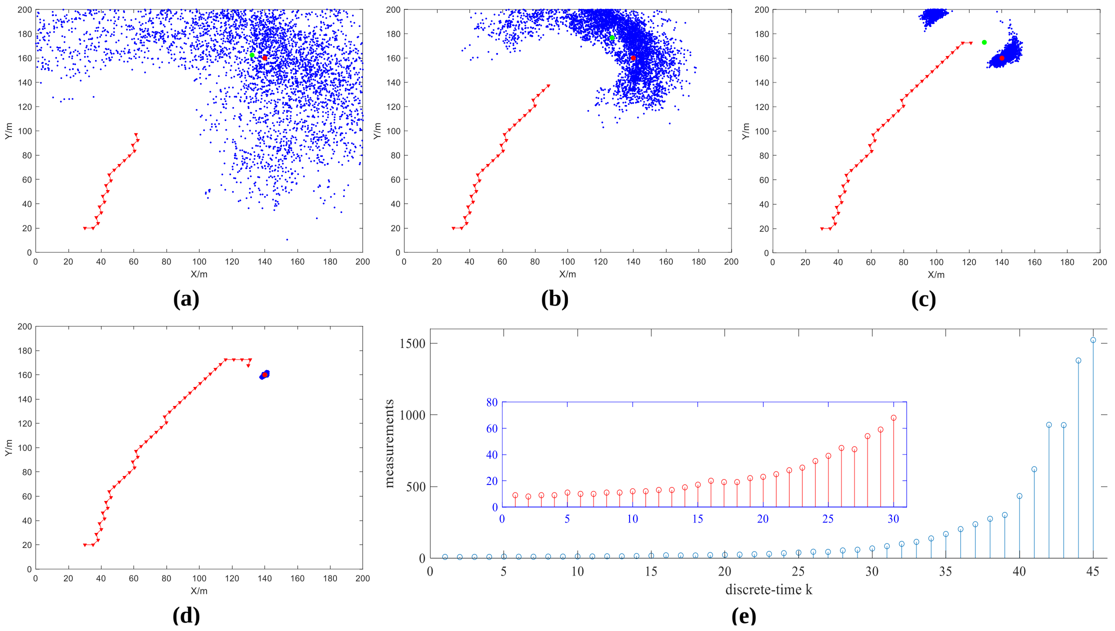

Figure S7. The estimation results of radioactive sources when the number of measurements $N = 20$ and angular velocity $\omega = 0.9rad/s$ (**a**) $k = 20$, (**b**) $k = 30$, (**c**) $k = 40$, (**d**) $k = 45$. Figure (**e**) shows the measured value z_k obtained during the search of the radioactive source.

Supplementary Materials 8: Real experiment results

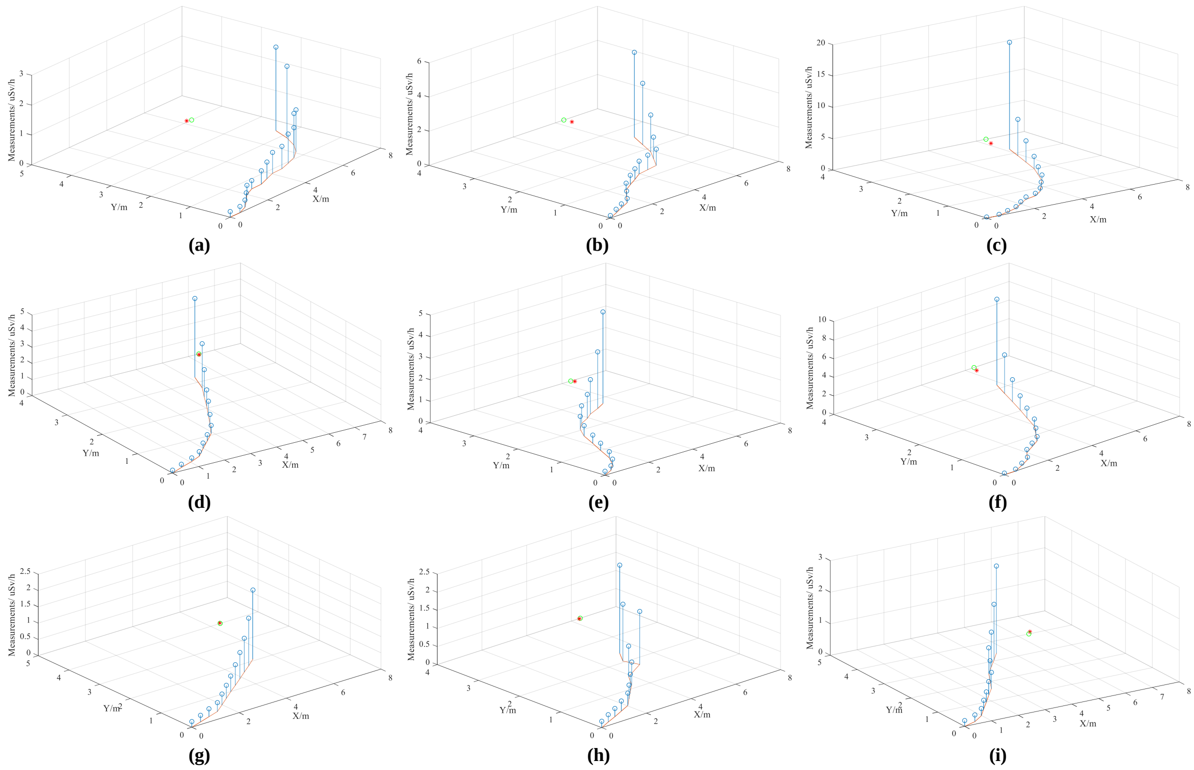

Figure S8. Real experiment results. When angular velocity $\omega = 0.1rad/s$, the results of(**a**) $N = 1, v = 0.1m/s$, (**b**) $N = 1, v = 0.15m/s$, (**c**) $N = 1, v = 0.2m/s$, (**d**) $N = 2, v = 0.1m/s$, (**e**) $N = 2, v = 0.15m/s$, (**f**) $N = 2, v = 0.2m/s$, (**g**) $N = 3, v = 0.1m/s$, (**h**) $N = 3, v = 0.15m/s$, (**i**) $N = 3, v = 0.2m/s$. In the figure, the green circle indicates the true position of the radioactive source, the red "*" indicates the estimated position of the radioactive source, the red line indicates the robot search path.
